# The Safety Profile of COVID-19 Vaccines in Patients Diagnosed with Multiple Sclerosis: A Retrospective Observational Study

**DOI:** 10.3390/jcm11226855

**Published:** 2022-11-21

**Authors:** Giorgia Teresa Maniscalco, Cristina Scavone, Annamaria Mascolo, Valentino Manzo, Elio Prestipino, Gaspare Guglielmi, Maria Luisa Aiezza, Santolo Cozzolino, Adele Bracco, Ornella Moreggia, Daniele Di Giulio Cesare, Antonio Rosario Ziello, Angela Falco, Marida Massa, Massimo Majolo, Eliana Raiola, Roberto Soprano, Giuseppe Russo, Giuseppe Longo, Vincenzo Andreone, Annalisa Capuano

**Affiliations:** 1 Multiple Sclerosis Regional Center, “A. Cardarelli” Hospital, 80131 Naples, Italy; 2 Neurological Clinic and Stroke Unit, “A. Cardarelli” Hospital, 80131 Naples, Italy; 3 Department of Experimental Medicine, University of Campania “Luigi Vanvitelli”, 80138 Naples, Italy; 4 Regional Center of Pharmacovigilance and Pharmacoepidemiology of Campania Region, 80138 Naples, Italy; 5 Pharmacy, “A. Cardarelli” Hospital, 80131 Naples, Italy; 6 Biotechnology Center, “A. Cardarelli” Hospital, 80131 Naples, Italy; 7 Healtcare Direction, “A. Cardarelli” Hospital, 80131 Naples, Italy; 8 General Direction, “A. Cardarelli” Hospital, 80131 Naples, Italy

**Keywords:** COVID-19, mRNA-based vaccine, safety, multiple sclerosis, AEFI, observational study

## Abstract

In the current COVID-19 pandemic, patients diagnosed with multiple sclerosis (MS) are considered to be one of the highest priority categories, being recognized as extremely vulnerable people. For this reason, mRNA-based COVID-19 vaccines are strongly recommended for these patients. Despite encouraging results on the efficacy and safety profile of mRNA-based COVID-19 vaccines, to date, in frail populations, including patients diagnosed with MS, this information is rather limited. We carried out a retrospective observational study with the aim to evaluate the safety profile of mRNA-based COVID-19 vaccines by retrieving real-life data of MS patients who were treated and vaccinated at the Multiple Sclerosis Center of the Hospital A.O.R.N. A. Cardarelli. Three-hundred and ten medical records of MS patients who received the first dose of the mRNA-based COVID-19 vaccine were retrieved (63% female; mean age: 45.9 years). Of these patients, 288 also received the second dose. All patients received the Pfizer-BioNTech vaccine. Relapsing-Remitting Multiple Sclerosis (RRSM) was the most common form of MS. The Expanded Disability Status Scale (EDSS) values were <3.0 in 70% of patients. The majority of patients received a Disease Modifying Therapy (DMT) during the study period, mainly interferon beta 1-a, dimethyl fumarate, and natalizumab and fingolimod. Overall, 913 AEFIs were identified, of which 539 were after the first dose of the vaccine and 374 after the second dose. The majority of these AEFIs were classified as short-term since they occurred within the first 72 h. The most common identified adverse events were pain at injection site, flu-like symptoms, and headache. Fever was reported more frequently after the second dose than after the first dose. SARS-CoV-2 infection occurred in 3 patients after the first dose. Using historical data of previous years (2017–2020), the relapses’ rate during 2021 was found to be lower. Lastly, the results of the multivariable analysis that assessed factors associated with the occurrence of AEFIs revealed a statistical significance for age, sex, and therapy with ocrelizumab (*p* < 0.05). In conclusion, our results indicated that Pfizer-BioNTech vaccine was safe for MS patients, being associated with AEFIs already detected in the general population. Larger observational studies with longer follow-up and epidemiological studies are strongly needed.

## 1. Introduction

Since the beginning of the COVID-19 outbreak, a range of repurposed drugs against this new infection have been investigated with the aim to fight one of the largest international health emergencies [1,2,3]. Following the identification of the genetic sequence of SARS-CoV-2, the virus responsible for COVID-19, an intense research program on potential vaccines started worldwide. To date (November 2022), six vaccines have obtained marketing approval from the European Medicines Agency (EMA), including two RNA vaccines (Pfizer-BioNTech and Moderna), two adenovirus vaccines (AstraZeneca and Janssen), and one recombinant, adjuvanted vaccine (Novavax) and one inactivated, adjuvanted (COVID-19 Vaccine Valneva). The Pfizer-BioNTech vaccine is a modified mRNA-based vaccine that encodes a S protein of the virus. The RNA of this vaccine is produced by in vitro transcription from the corresponding DNA that encodes a S protein of SARS-CoV-2, which is responsible of the virus’ attachment to the host’s cells [4]. The Moderna vaccine has similar characteristics. Both vaccines are given as two injections, usually into the muscle of the upper arm, 3 and 4 weeks apart for the Pfizer and Moderna vaccine, respectively. The efficacy of both vaccines was evaluated in thousands of people aged 16 and above who had no sign of previous infection, resulting in an almost 95% reduction in the number of symptomatic COVID-19 cases in people who received the vaccination [5,6]. Preliminary data on their safety profile showed that adverse events following immunization (AEFIs) with mRNA-based COVID-19 vaccines are generally mild and consist of injection site reactions, headache, and asthenia [7].

According to the COVID-19 vaccination program of the Italian Ministry of Health, patients diagnosed with multiple sclerosis (MS) are considered to be one of the highest priority categories, being recognized as extremely vulnerable people. For this reason, mRNA-based COVID-19 vaccines are strongly recommended for these patients [8]. MS is an inflammatory-mediated demyelinating disease of the Central Nervous System caused by an immune dysregulation associated with genetic and environmental factors, including Epstein–Barr virus infection and cigarette smoking [9,10]. Worldwide, almost 2.5 million people are diagnosed with MS, mainly women aged 20–40 years [11]. Despite encouraging results on the efficacy and safety profile of mRNA-based COVID-19 vaccines, to date, in frail populations, including patients diagnosed with MS, this information is rather limited [12]. In addition, as both vaccines received conditional marketing authorization, the companies that market them must continue to provide results from the main clinical trials, which are still ongoing. These results, together with those provided by any other clinical study, will provide new data on the vaccine’s long-term safety and benefit in the general population, including frail patients.

Based on these considerations, we carried out a retrospective observational study with the aim to evaluate the safety profile of COVID-19 vaccines by retrieving real-life data of MS patients who were treated and vaccinated at the Multiple Sclerosis Center of the Hospital A.O.R.N. A. Cardarelli. Specifically, we aimed to evaluate the safety profile of mRNA-based COVID-19 vaccines in MS patients, in terms of any AEFI occurring in the short- and long-term after each dose of these vaccines; identify any possible correlations between AEFIs and MS patients’ demographic characteristics, clinical status, and their disease modifying therapies (DMTs); identify cases of relapses following mRNA-based COVID-19 vaccines and compare relapses’ rate with those of previous years in the same population.

## 2. Methods

### 2.1. Study Design

This is a retrospective observational study carried out with data retrieved from medical records of SM patients treated and vaccinated at the MS Centre of the Cardarelli Hospital (Naples, South of Italy) for the period from March 2021 to September 2021 to assess the safety of mRNA-based COVID-19 vaccines in this population.

### 2.2. Demographic and Clinical Data Collection

The following data were retrieved: demographic data (MS diagnosis, age, gender); mRNA-based COVID-19 vaccine (date of vaccination, type of vaccine, vaccine batch); medical and clinical history, DMTs; all AEFIs that occurred after vaccination, in terms of number, time of occurrence and type (where available); patients’ general clinical course.

### 2.3. AEFIs Data Collection

According to the European and Italian rules on the management of ICSRs [13,14], when an AEFI likely associated with the COVID-19 vaccine was identified from the medical records, the suspected reporting form established by the Italian Medicine Agency (AIFA) was filled in, if not previously performed. Then, the suspected reporting form was sent to the qualified person responsible for pharmacovigilance who recorded the ADR report into the Italian spontaneous reporting database (RNF).

We carried out a descriptive analysis of all identified AEFIs in terms of time of occurrence and Preferred term (PT), with a focus on MS relapses. Regarding the time of occurrence, AEFIs were classified as short-term when they occurred within 72 h the first and second dose of the vaccine and as long-term when they occurred within 20 days the first and second dose of the vaccine.

### 2.4. Statistical Analysis

Categorial variables were described as frequency and percentage, while continuous variables were reported by their median and mean. For the evaluation of the relapse rate following the vaccine, we retrieved historical data of previous years (from March to September during years 2017–2020) referred to the same population. This comparison was made in order to detect any differences with previous years’ relapses considering the effects deriving both from the social distancing measures adopted across Italian territory and the immunization with the mRNA-based COVID-19 vaccine. We used a multivariable model (multiple logistic regression) to check for a difference of total AEFIs that occurred both after the first and the second doses, adjusted for age, sex, disease duration, Expanded Disability Status Scale (EDSS) and DMTs. Beta coefficients were calculated in order to compare the strength of the effect of each individual independent variable to the dependent variable. The EDSS is a method of quantifying disability in MS patients and monitoring changes in the level of disability over time.

### 2.5. Ethics

According to the Italian law, the retrospective study was notified to the Ethic Committee of A.O.R.N. A. Cardarelli/Santobono-Pausilipon. No written informed consent was necessary for the study conduction based on its retrospective nature.

## 3. Results

We retrieved 310 MS patients (63% female; mean age: 45.9 years) who received the first dose of the mRNA-based COVID-19 vaccine. Of these patients, 288 also received the second dose. All patients received the Pfizer-BioNTech vaccine. Relapsing-Remitting Multiple Sclerosis (RRSM) was the most common form of MS, which was identified in almost 87% of patients (n = 270), while almost 10% of patients were diagnosed with Secondary Progressive Multiple Sclerosis (SPSM) and 2.6% with Primary Progressive Multiple Sclerosis (PPSM). EDSS values were <3.0 in 70% of patients, equal to 3.5–5.5 in 22% of patients and ≥6.0 in 7% of patients. Approximately 10% of patients had a previous COVID-19 infection before the enrolment in our study (Table 1). The majority of retrieved patients (93.9%) were receiving a DMT during the study period. In particular, those prescribed in more than 10% of patients were interferon beta 1-a (19.7%), dimethyl fumarate (15.2%), and natalizumab (15.2%) and fingolimod (11.3%).

Overall, 913 AEFIs were identified during the study period, of which 539 were after the first dose of the vaccine and 374 after the second dose. The majority of these AEFIs were classified as short-term since they occurred within the first 72 h (86.5% short-term vs. 13.5% long-term). This trend was confirmed after the first and second doses (Table 2).

**Figure 1:**
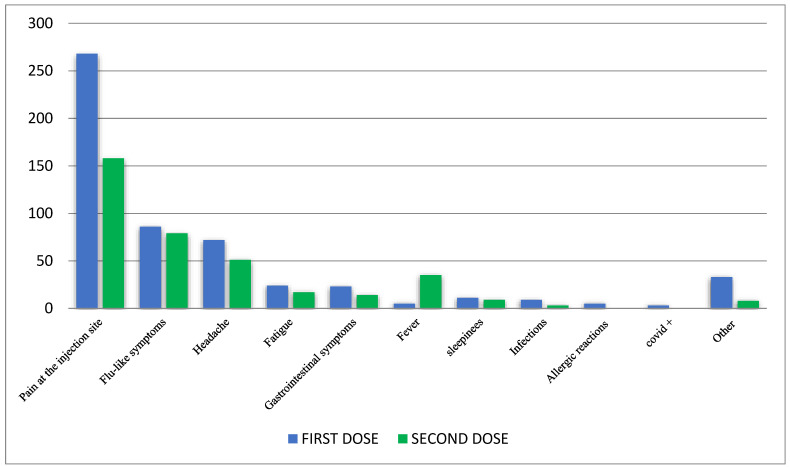
Distribution of Adverse Events Following Immunization (AEFIs) by Preferred Term (PT).

The most common reported PTs were pain at injection site (n = 426; 46.7%), flu-like symptoms (n = 165; 18.1%), and headache (n = 123; 13.5%). No substantial differences were found in terms of PTs distribution by first and second dose (Figure 1). Fever was reported more frequently after the second dose than after the first dose. SARS-CoV-2 infection occurred in 3 patients after the first dose.

Regarding the relapse rate, a transient increase in MS symptoms following vaccination (pseudo-relapse) was reported in 6 patients out of 310 (1.9%), of which 2 were after the first dose (0.6% of vaccinated patients) and 4 after the second dose (1.4% of vaccinated patients). During the study period (March 2021–September 2021), relapses were identified in 5 patients, of which 3 were after first dose of the vaccine (0.9% of patients) and 2 after the second dose (0.7% of patients).

**Figure 2:**
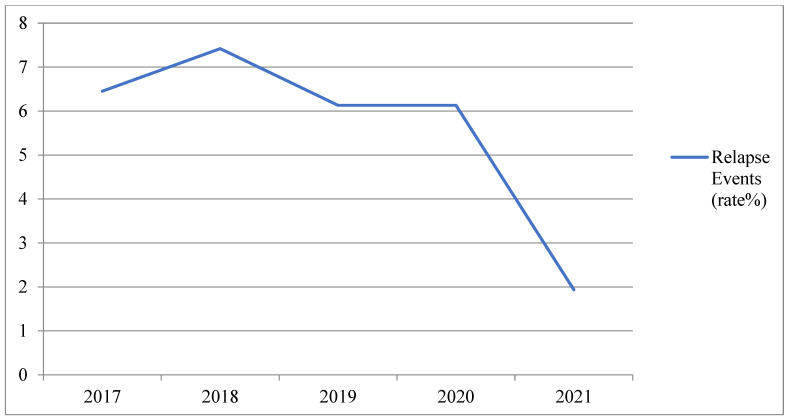
Trend of Relapses (%) during years 2017–2021 (periods considered: March–September).

Using historical data from previous years (2017–2020), the relapse rates were found to be 6.1%, 6.1%, 7.4%, and 6.4%, respectively (Figure 2).

Lastly, the results of the multivariable analysis that assessed factors associated with the occurrence of AEFIs revealed a statistical significance for age, sex, and therapy with ocrelizumab (*p* < 0.05) (Table 3), suggesting that AEFIs were less common in young patients (negative β coefficient = −0.02, *p* = 0.03), while they were more common in female patients (positive β coefficient = 0.361, *p* = 0.03) and in patients who were receiving ocrelizumab (positive β coefficient = 0.477, *p* = 0.022).

## 4. Discussion

We carried out a study with the aim to evaluate the safety profile of mRNA-based vaccine against COVID-19 in patients diagnosed with MS. Similar to any other medicine, vaccines can be associated with the occurrence of adverse events. An AEFI is defined as “*any untoward medical occurrence which follows immunization and which does not necessarily have a causal relationship with the usage of the vaccine*” [15]. AEFIs also include those events associated with vaccination, patient anxiety-related responses, and product quality defect [16,17,18]. Currently, AEFIs associated with mRNA COVID-19 vaccines in real-life settings are consistent with those already mentioned in the summary of product characteristics of these vaccines. For both vaccines, the most reported AEFIs are injection site pain, fever, asthenia, and muscle aches. Headache, paresthesia, dizziness, drowsiness, taste disturbances, nausea, and abdominal pain have also been observed with both vaccines. These AEFIs are generally not serious and resolve spontaneously. In line with our results, fever is reported more frequently after the second dose. Events occur mostly on the same day as vaccination or the day after [19].

We reported the results related to 310 MS patients who received the first dose of the Pfizer-BioNTech mRNA-based vaccine and 288 who received the second dose of the same vaccine. In our study, the mean age of patients was 45.9 years, and almost 63% were female. RRSM was the most common form of MS, while the most commonly prescribed DMTs were interferon, dimethyl fumarate, natalizumab, and fingolimod. These demographic characteristics were expected. Indeed, as we previously reported [20,21], MS shows the highest prevalence in the age group 35–64 years and with a well-known female predominance (the prevalence ratio of MS of women to men is 2.3–3.5:1) [22,23]. With regard to the most commonly prescribed DMTs, in our opinion the distribution in drugs’ utilization could be related to the variegate population included in our study mainly in terms of age and MS type.

Other international research groups carried out clinical studies with the aim to evaluate the safety profile of COVID-19 vaccines in MS patients. For instance, Lotan I et al. carried out a survey among 425 MS patients with questions related to their general demographic and disease-related characteristics and the safety profile of COVID-19 vaccines. Out of the 425 patients, 262 completed the questionnaire, of which 239 had received the Pfizer-BioNTech vaccine. Almost 56% of patients (n = 136) experienced AEFIs, while 15% of them (n = 36) reported new or worsening neurological symptoms following the vaccination (mainly sensory disturbances). In line with our results, AEFIs commonly occurred within the first 24 h after vaccination and resolved within 3 days [24]. The safety profile of the Pfizer-BioNTech vaccine was evaluated in another observational study that enrolled 555 MS patients who received the first dose of the vaccine (of these, 435 received the second dose). This study was carried out in one clinical Centre in Israel where all patients received the Pfizer-BioNTech vaccine. In line with our findings, the PTs most commonly reported were pain at the injection site, fatigue, and headache, while the rate of patients with relapse was 2.1% and 1.6% following the first and second doses, respectively. The comparison of these rates with those from previous years highlighted no differences, although the short follow-up period could have resulted in lower relapse rates. As in our cohort, also in this study, three cases of COVID-19 infection were identified after the first dose [25].

Compared with previous years, we found a reduced relapse rate among MS patients who had received the vaccine during the study period [6 of 310 in 2021 (1.9%) vs. 19 of 310 in 2020 and 2019 (6.1%) vs. 23 of 310 in 2018 (7.4%) vs. 20 of 310 in 2017 (6.4%)]. In our opinion, this finding could be the result of the social distancing measures imposed by the Italian government to vulnerable patients, such as MS patients, which could have positively affected the risk of infection in general, leading to a reduction in relapses too. Indeed, the role of viral respiratory tract infections in increasing the risk of MS relapses [26,27,28,29] is widely recognized, especially considering that following a viral infection the activation of proinflammatory patterns, such as the host’s T-cells, proinflammatory cytokines, and tumor necrosis factor (TNF)-α, increases the blood-brain barrier (BBB) permeability, allowing for transmigration of those molecules and promoting central nervous system inflammation [30]. In this respect, Landi et al. carried out a survey among MS patients followed at the MS center of Tor Vergata University hospital in Italy with the aim to explore adherence to social distancing habits. The results revealed that patients demonstrated good adherence to social distancing and use of protection equipment [31]. In addition, even though few cases of MS relapses following COVID-19 vaccines could be found in the literature [32,33,34,35], as recently reported by Di Filippo et al. [36], currently the evidence of a possible association between these vaccines and MS activity is still debated. Indeed, although mRNA-based COVID-19 vaccines might elicit a strong T and B cells response leading to the development of autoimmune processes [37,38], since systemic infections, such as COVID-19, can worsen MS, the vaccination can be able to reduce the risk of relapses by dropping the risk of infections [25]. Thus, no confirmed association between the Pfizer/BioNTech vaccine and the short-term risk of clinical reactivation in MS exists [36].

Finally, with regard to the results of the multivariable analysis, we found that AEFIs following the mRNA-based COVID-19 vaccine were less common in young patients, while they were more common in female patients and in patients who were receiving ocrelizumab. Contrary to our findings, Achiron et al. found a mild increase in the rate of AEFIs in younger patients and in those treated with immunomodulatory drugs [25]. With regard to the results of statistical analysis reporting a female predominance, many studies highlighted how differences in immunological, hormonal, and genetic mechanisms could explain disparities between men and women in vaccines’ response (both in term of efficacy and safety) [18,39,40], leading to higher rate of AEFIs in female patients [39]. Indeed, compared with men, women typically develop higher antibody responses to vaccines, experiencing more local and systemic adverse reactions [40].

With regard to the association with ocrelizumab, to our knowledge, no other studies have evaluated the safety profile of mRNA vaccines in MS patients receiving this drug. As we recently reported [41], it is known that some DMTs might induce immunomodulation through lymphocyte depletion, leading to different protective humoral and cellular responses to COVID-19 vaccines. For instance, rituximab, ocrelizumab, and fingolimod have been associated with changes in SARS-CoV-2 IgG antibody production in MS-treated subjects [25,42,43]. In this respect, as reported by Zabalza et al., 2021, less than 20% of MS patients treated with ocrelizumab generate an antibody response when naturally infected by COVID-19 [44]. Further studies evaluating humoral and cellular immune response to COVID-19 vaccines and their safety profiles among MS patients undergoing different DMTs are strongly needed.

## 5. Strengths and Limitations

Our study has several limitations, including its retrospective and monocentric nature and small sample size. Thus, our findings regarding the safety profile of the Pfizer-BioNTech vaccine in MS patients should be considered exploratory since we can’t exclude the lack of important clinical data which might have led to different conclusions.

Nevertheless, to our knowledge, this is the first Italian study carried out in a real-life context among MS patients with the aim to evaluate the safety profile of mRNA-based COVID-19 vaccines. Even though the study was carried out in a single clinical center, it covers a population of approximately 800 MS patients. In addition, data collection and analysis have been performed by a multidisciplinary team of neurologists, pharmacologists, statisticians, and data managers. Lastly, using historical data from 2017 to 2020, we were able to make a comparison in term of relapses’ rate and to provide possible explanations underlying the lower relapses’ rate that we found during the period March–September 2021.

## 6. Conclusions

In conclusion, our results indicated that the Pfizer-BioNTech vaccine was safe for MS patients, being associated with AEFIs already detected in the general population. In addition, during the study period a reduced rate of MS relapse was found, which in our opinion could be related to the preventing measures introduced by the Italian government. The results of statistical analysis suggested that AEFIs might be more common in young and female MS patients and in those treated with ocrelizumab. While a higher rate of vaccine-induced AEFIs in young and female patients can be expected, no data are currently available regarding the association with ocrelizumab treatment. Larger observational studies with longer follow-up and epidemiological studies are strongly needed in order to collect more data on the safety profile of COVID-19 vaccines in the frail population [45,46,47].

## Figures and Tables

**Table 1:** Clinical and demographic variables of patients with multiple sclerosis who received COVID-19 vaccination.

Study Cohort	First Vaccine Dose	Second Vaccine Dose
Total patients	310	288
Gender, n (%)		
Female	195 (62.9)	179 (62.2)
Male	115 (37.1)	109 (37.8)
Mean age (years)	45.9	45.8
Disease duration, years (mean)	11.8	11.6
Median (years)	10.1	10.0
MS type, n (%)		
RRMS	270 (87.1)	251 (87.1)
SPMS	32 (10.3)	29 (10.1)
PPMS	8 (2.6)	8 (2.8)
Disability by EDSS, n (%)		
≤3.0	219 (70.6)	209 (72.6)
3.5–5.5	69 (22.3)	59 (20.5)
≥6.0	22 (7.1)	20 (6.9)
SARS-CoV-2 infection before vaccination, n (%)	34 (10.7)	19 (6.6)
DMTs, n (%)		
Interferon Beta 1-a	61 (19.7)	55 (19.2)
Glatiramer acetate	20 (6.5)	19 (6.7)
Teriflunomide	28 (9.0)	26 (9.0)
Dimethyl fumarate	47 (15.2)	45 (15.6)
Fingolimod	35 (11.3)	35 (12.1)
Natalizumab	47 (15.2)	43 (14.9)
Cladribine	17 (5.5)	12 (4.2)
Ocrelizumab	23 (7.4)	22 (7.7)
Interferon Beta 1-b	7 (2.3)	7 (2.4)
Azathioprine	1 (0.3)	1 (0.3)
Methotrexate	3 (0.9)	3 (1.0)
Rituximab	2 (0.6)	2 (0.7)
Untreated	19 (6.1)	18 (6.2)

RRMS: Relapsing Remitting Multiple Sclerosis; SPMS: Secondary Progressive Multiple Sclerosis; PPMS: Primary Progressive Multiple Sclerosis; EDSS: Expanded Disability Status Scale; DMTs: Disease Modifying Therapies.

**Table 2:** Distribution of Adverse Events Following Immunization (AEFIs) by time of occurrence (short- and long-term) and vaccine’s dose.

	Total	First Vaccine Dose	Second Vaccine Dose
Study population	310	310	288
All AEFIs, n	913	539	374
Short-term AEFIs, n (%)	790 (86.5)	438 (81.3)	352 (94.1)
Long-term AEFIs, n (%)	123 (13.5)	101 (18.7)	22 (5.9)

**Table 3:** Multivariable analysis assessing factors associated with Adverse Events Following Immunization (AEFIs).

Variable	Multivariable Analysis	
	Beta Coefficients (SE)	*p* Value
EDSS	−0.08 (0.08)	0.30
Disease duration	0.02 (0.01)	0.10
Age	−0.02 (0.01)	0.03
Sex (female vs. male)	0.361 (0.09)	<0.001
DMTs		
No therapy	Ref	Ref
Interferon Beta 1-a	0.263 (0.185)	0.156
Interferon Beta 1-b	−0.361 (0.367)	0.326
Glatiramer Acetate	0.071 (0.228)	0.756
Teriflunomide	0.118 (0.213)	0.579
Dimethyl fumarate	0.094 (0.193)	0.626
Natalizumab	0.068 (0.197)	0.730
Fingolimod	0.099 (0.202)	0.625
Ocrelizumab	0.477 (0.208)	0.022
Cladribine	0.101 (0.236)	0.669
Other treatment	−0.171 (0.357)	0.631

EDSS: Expanded Disability Status Scale; DMT: Disease Modifying Therapies; SE: Standard Error.

## Data Availability

The data presented in this study are available on request from the corresponding author. The data are not publicly available due to privacy and ethical reasons.
